# Recombinant erythropoietin does not augment hypothermic white matter protection after global cerebral ischaemia in near-term fetal sheep

**DOI:** 10.1093/braincomms/fcab172

**Published:** 2021-07-29

**Authors:** Guido Wassink, Joanne O Davidson, Alyssa Crisostomo, Kelly Q Zhou, Robert Galinsky, Simerdeep K Dhillon, Christopher A Lear, Laura Bennet, Alistair J Gunn

**Affiliations:** 1 Department of Physiology, The University of Auckland, Auckland, New Zealand; 2 The Ritchie Centre, Hudson Institute of Medical Research, Victoria, Australia

**Keywords:** cerebral ischaemia, erythropoietin, hypothermia, white matter, foetal sheep

## Abstract

Therapeutic hypothermia for hypoxic-ischaemic encephalopathy provides partial white matter protection. Recombinant erythropoietin reduces demyelination after hypoxia-ischaemia, but it is unclear whether adjunct erythropoietin treatment can further improve outcomes after therapeutic hypothermia. Term-equivalent fetal sheep received sham-ischaemia (*n* = 9) or cerebral ischaemia for 30 min (ischaemia-vehicle, *n* = 8), followed by intravenous infusion of recombinant erythropoietin (ischaemia-Epo, *n* = 8; 5000 IU/kg bolus dose, then 833.3 IU/kg/h), cerebral hypothermia (ischaemia-hypothermia, *n* = 8), or recombinant erythropoietin plus hypothermia (ischaemia-Epo-hypothermia, *n* = 8), from 3 to 72 h post-ischaemia. Foetal brains were harvested at 7 days after cerebral ischaemia. Ischaemia was associated with marked loss of total Olig2-positive oligodendrocytes with reduced density of myelin and linearity of the white matter tracts (*P* < 0.01), and microglial induction and increased caspase-3-positive apoptosis. Cerebral hypothermia improved the total number of oligodendrocytes and restored myelin basic protein (*P* < 0.01), whereas recombinant erythropoietin partially improved myelin basic protein density and tract linearity. Both interventions suppressed microgliosis and caspase-3 (*P* < 0.05). Co-treatment improved 2′,3′-cyclic-nucleotide 3′-phosphodiesterase-myelin density compared to hypothermia, but had no other additive effect. These findings suggest that although hypothermia and recombinant erythropoietin independently protect white matter after severe hypoxia-ischaemia, they have partially overlapping anti-inflammatory and anti-apoptotic effects, with little additive benefit of combination therapy.

## Teaser

Wassink et al. report that in near-term fetal sheep induced hypothermia and high-dose recombinant erythropoietin infusion started 3 h after cerebral ischaemia and continued for 3 days, independently protected white matter after 7 days recovery. However, there were overlapping anti-inflammatory and anti-apoptotic effects, with little additive benefit of combination therapy.

## Graphical Abstract

**Graphical Abstract:**
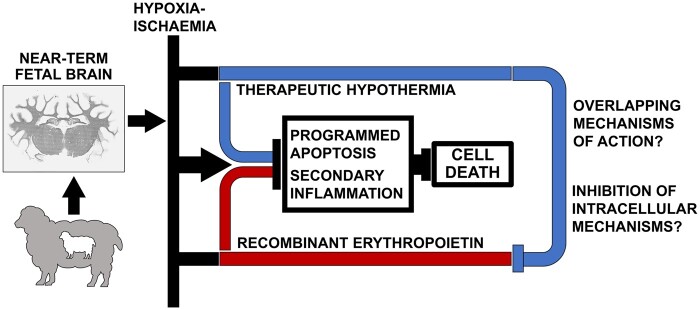

## Introduction

Perinatal hypoxic-ischaemic encephalopathy (HIE) is a significant contributor to neonatal death and permanent neurodevelopmental disabilities around the world.^1^ Therapeutic hypothermia is standard care in the developed world, but only partially improves outcomes, so that many children survive with disability, including reduced cognitive and motor performance even in survivors without cerebral palsy.^2^^,^^3^ Recent preclinical and clinical studies demonstrate that current hypothermia protocols are essentially optimal,^4^ and so new strategies are needed to improve outcomes.

Historically, research into neuroprotection focussed on grey matter. Importantly, white matter damage is common after HIE in term and near-term infants and highly associated with adverse neurodevelopmental outcomes.^5–7^ Interestingly, in a cohort study, even isolated cortical white matter injuries correlated with neurological problems at 2 years of age.^8^ In preclinical studies of therapeutic hypothermia in near-term fetal sheep, there is evidence that the window of opportunity for white matter protection is relatively shorter than for neuronal protection, such that hypothermia started up to 5.5 h after cerebral ischaemia partially improved neuronal survival,^9^ but did not improve survival of oligodendrocytes.^10^^,^^11^ Thus, white matter damage despite therapeutic hypothermia may be an important target.

Recombinant erythropoietin (rEpo), a pleiotropic trophic factor, has potent anti-apoptotic, anti-excitotoxic and anti-inflammatory effects after perinatal hypoxia-ischaemia (HI) and stroke.^12–14^ Importantly, it promotes maturation and differentiation of oligodendrocytes.^15^ Critically, in neonatal P7 rats, delayed rEpo attenuated white matter lesions, increased neuro- and gliogenesis and development of oligodendrocytes, and improved neurobehavioral outcome after 2 weeks,^16^ suggesting that it may have a particular role in alleviating white matter injury.

There is only limited, conflicting evidence for whether adjunct rEpo treatment combined with therapeutic hypothermia can alleviate white matter injury. For example, in neonatal rats, concurrent treatment with rEpo and hypothermia initiated after HI did not further improve white matter damage or sensorimotor scores, compared to hypothermia alone.^17^ In non-human primates, repeated rEpo injections starting 30 min after birth asphyxia plus cooling reduced cerebral palsy with improved diffusivity and fractional anisotropy values of the corpus callosum, anterior and posterior limbs of the internal capsule, and other white matter tracts on diffusion tensor imaging.^18^^,^^19^ However, outcomes after combined treatment were not statistically different from hypothermia alone. In piglets, hypothermia from 1 to 13 h after HI plus rEpo (3000 IU/kg) injections at 1, 24 and 48 h was associated with more rapid EEG recovery and improved oligodendrocyte survival.^20^ It is unknown whether this would have been seen with a clinical protocol of prolonged hypothermia. By contrast, a small phase-II trial in HIE infants that received adjunct rEpo suggested fewer subcortical and cerebellar lesions on MRI compared to hypothermia-treated infants,^21^ but no change in global white matter injury or neurological outcomes.

Thus, in this study, we tested the hypothesis that a prolonged, high-dose infusion of rEpo from 3 to 72 h after cerebral ischaemia, that achieved sustained plasma levels consistent with those required for neuroprotection in rats^22^^,^^23^; would reduce oligodendroglial cell loss and demyelination at 7 days after cerebral ischaemia in term-equivalent fetal sheep. Furthermore, we tested whether the combination of the rEpo infusion plus a standard hypothermia protocol over the same interval would augment white matter protection with hypothermia.

## Materials and methods

### Ethical approval

All procedures were approved by the Animal Ethics Committee of The University of Auckland under the provisions of the New Zealand Animal Welfare Act, and followed the National Institutes of Health directives (NIH Publication 8023) and Code of Ethical Conduct from the Ministry of Primary Industries, Government of New Zealand. This manuscript conforms with the ARRIVE guidelines for designing and reporting animal research.^24^

### Experimental preparation

Forty-one time-mated Romney/Suffolk fetal sheep were instrumented at 124.5 ± 0.3 days (d) gestation (term = 147 days).^14^ Food, but not water was withdrawn 12–18 h before the procedure. Ewes received oxytetracycline (20 mg/kg, Phoenix Pharm, Auckland, New Zealand) 30 min before surgical procedures. Anaesthesia was induced via intravenous (i.v.) injection with Propofol (5 mg/kg, AstraZeneca Limited, Auckland, New Zealand), followed by 2–3% isoflurane (Medsource, Ashburton, New Zealand) in oxygen. Anaesthesia and physiological parameters were monitored by trained staff, and ewes received i.v. saline to maintain fluid balance.

Surgical procedures were undertaken using sterile techniques, as described.^14^ The foetus was exposed by laparotomy. The right fetal brachial vein and both brachial arteries were catheterized for pre-ductal blood sampling, and to record mean arterial blood pressure (MAP), and infuse drugs. Electrodes (Cooner Wire Company, Chatsworth, CA, USA) were implanted across the chest to measure fetal heart rate (FHR). The vertebral–occipital anastomoses were ligated, and inflatable occluders were secured around the common carotid arteries.^14^ An ultrasonic transit-time flow probe (Transonic Systems Inc., Ithaca, NY, USA) was placed around the carotid artery. EEG electrodes (Cooner Wire Company) were positioned on the parietal dura (10 mm and 20 mm anterior to bregma, and 10 mm lateral), with a reference sutured on the occiput. Electrodes (Cooner Wire Company) were placed 5 mm lateral to the EEG electrodes to record cortical impedance, as an index of cell swelling.^25^

Thermal probes (Mallinckrodt Pharmaceuticals, Chesterfield, UK) were inserted into the oesophagus, to measure body core temperature and over the parasagittal dura (30 mm anterior to bregma) to measure extradural temperature. A silicone cooling coil (Degania Silicone Ltd, Israel) was secured over the cranium. The uterine incision was closed and gentamicin sulphate (80 mg, Pfizer, Auckland, New Zealand) was administered into the amniotic cavity. The maternal laparotomy incision was sutured and infiltrated with 0.25% bupivacaine plus adrenaline (AstraZeneca Ltd). All leads were tunnelled through the maternal flank and a saphenous vein was catheterized for post-operative care. If twins were present, only a single foetus was instrumented. Foetuses whose weight was <10th percentile for age were excluded.

### Post-operative care

Ewes were kept together in individual metabolic crates with ad libitum access to water and food. The animal housing was climate-controlled (16 ± 1°C, humidity 50 ± 10%), and operated on a 12 h day/night cycle. Ewes were given i.v. gentamicin (40 mg for 2 days, Pfizer) and benzylpenicillin (600 mg for 4 days, Onelink, Auckland, New Zealand). Fetal wellbeing was monitored by blood gas sampling and FHR and MAP monitoring. Vascular lines were infused with heparinized saline (0.15 ml/h of 20 IU heparin/ml).

### Experimental data

Experiments were undertaken at 128.9 ± 0.1 days gestation. Physiological/neurophysiological data were recorded from 24 h before until 168 h after carotid occlusion or sham occlusion, using custom processing hardware and Labview-based acquisition software (National Instruments). Preductal fetal blood samples were analysed for pH, blood gases (Radiometer, Auckland, New Zealand), glucose and lactate (YSI model 2300, Yellow Springs, OH, USA), and erythropoietin levels. All foetuses had baseline physiological and biochemical parameters that were appropriate for gestational age.^14^

### Experimental protocols

Foetuses were randomized by computer programme to receive sham-ischaemia (*n* = 9), ischaemia-vehicle (*n* = 8), ischaemia-Epo (*n* = 8), ischaemia-hypothermia (*n* = 8), or ischaemia-Epo-hypothermia (*n* = 8), using a random block design. Global cerebral ischaemia for 30 min was induced by inflating both carotid occluders, and confirmed by suppression of EEG power and a progressive increase in cortical impedance. Occluder cuffs were not inflated in sham-ischaemia animals. All experiments started at 09:00 h. In the hypothermia groups, cerebral cooling was started 3 h post-ischaemia and continued until 72 h, by connecting the cooling cap to a water circulator placed in a refrigerated water bath (Grant Instruments Ltd, Cambridgeshire, UK). Fetal extradural temperature was titrated to 31–33°C in the first 3 h of hypothermia. Cold water was not circulated in normothermia animals.

Foetuses received either rEpo (Janssen-Cilag Ltd, Auckland, New Zealand) dissolved in vehicle or isotonic saline with 0.1% bovine serum albumin (vehicle), i.v. starting 3 h post-reperfusion and continued until 72 h after cerebral ischaemia. Based on an estimated fetal weight of 4 kg based on historical records, foetuses were given a loading bolus of 5000 international units (IU) of rEpo/kg, followed with a constant infusion of 833.3 IU/kg/h. Control animals received an identical volume of vehicle. Experiments were terminated 7 days after cerebral ischaemia. Animals were killed with sodium pentobarbitone (9 g, i.v. to the ewe; Pentobarb 300, Provet, Auckland, New Zealand). Fetal organs were weighed and brains collected.

### Immunohistochemistry

Fetal brains were perfusion-fixed in situ using heparinized saline and 10% phosphate buffered formalin, post-fixed for 4–5 days, and embedded using a standard paraffin preparation. 10 µm thickness coronal sections from the level of the dorsal hippocampus (17 mm anterior to stereotaxic zero) were obtained using a microtome (Bio-Strategy Ltd, Auckland, New Zealand). Tissues were twice dewaxed in xylene (15 min each), rehydrated in ethanol (100%, 95% and 70%, 5 min each) and washed thrice in 0.1 mol/l phosphate buffered saline (PBS; 5 min each). Antigens were retrieved in 0.01 mol/l citrate buffer with an automated pressure cooker (Aptum Biologics Ltd, Southampton, United Kingdom) and endogenous peroxidase was quenched for 30 min by incubation in 1.0% H_2_O_2_ in PBS for oligodendrocyte transcription factor 2 (Olig2, labels all lineage stages) or methanol for myelin basic protein (MBP), 2′,3′-cyclic nucleotide 3′-phosphodiesterase (CNPase), ionized calcium-binding adapter molecule-1 (Iba1), glial fibrillary acidic protein (GFAP), proliferation marker Ki67 and cysteine-aspartic acid protease-3 (caspase-3). Non-specific antigen binding was blocked using 3% normal goat serum (NGS, Life Technologies Ltd, Auckland, New Zealand) in PBS for 1 h at room temperature.

Immunohistochemistry was performed using rabbit anti-Olig2 (AB109186, Abcam), mouse anti-CNPase (AB6319, Abcam), mouse anti-MBP (MAB381, Merck KGaA, Auckland, New Zealand), rabbit anti-Iba1 (AB178680, Abcam), rabbit anti-GFAP (AB68428, Abcam), mouse anti-Ki67 (M724001, Agilent Technologies, Mulgrave, Victoria, Australia) or rabbit cleaved caspase-3 (CTE9661S, Thermo Fisher Scientific Inc., Auckland, New Zealand) diluted 1:200 in 3% NGS-PBS, overnight at 4°C. Sections were then labelled with 1:200 goat anti-rabbit (Olig2, Iba1, GFAP and Caspase-3) or goat anti-mouse biotin-conjugated IgG (CNPase, MBP and Ki67; In Vitro Technologies, Auckland, New Zealand) in 3% NGS-PBS, overnight at 4°C. Slides were incubated in 1:200 ExtrAvidin-Peroxidase (Sigma-Aldrich, Auckland, New Zealand) in 3% NGS-PBS for 2 h at room temperature, and reacted in diaminobenzidine solution (Sigma-Aldrich). This reaction was stopped in PBS, and slides were dehydrated and DPX mounted (Scharlab, Barcelona, Spain).

**Figure 1:**
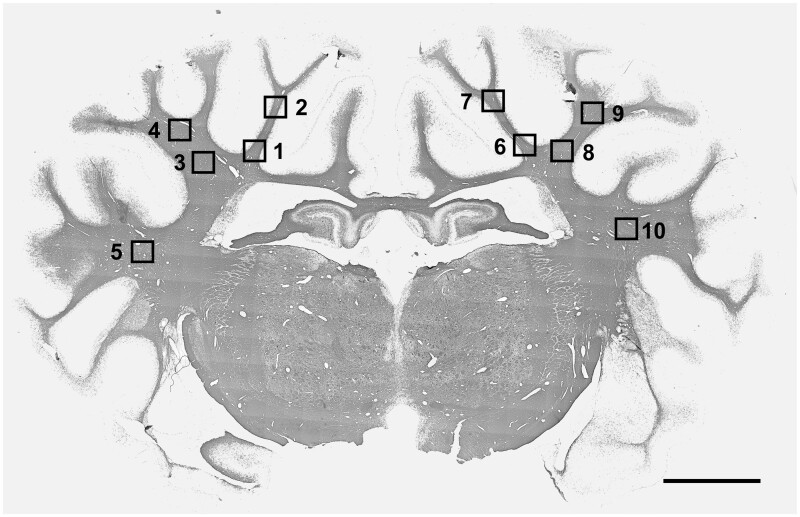
**Photomicrographs showing the fields of view used for analysis of white matter.** Photomicrographs showing the fields of view used for immunohistological analysis in the periventricular, parasagittal and lateral white matter. For sections taken at 17 mm anterior to stereotactic zero, one field in the periventricular white matter (squares 1 & 7), and one field in the corona radiata of the first and second parasagittal gyri (2, 4, 8, 10), in both hemispheres, were used for assessment. For Olig2-, 2′,3′-Cyclic-nucleotide 3'-phosphodiesterase (CNPase)- and myelin basic protein (MBP)-labelled brain sections, an additional field was assessed in the upper white matter tract of the first and second parasagittal gyrus (3, 5, 9, 11) and the lateral white matter (6, 12). Section stained with MBP. Magnification ×1. Scale bar is 5 mm.

White matter changes were quantified on images obtained at ×20 magnification on a Nikon Eclipse 80i microscope with a DS-Fi1-U3 camera, using NIS Elements Basic Research 5.1 (Nikon Instruments, Melville, NY, USA), on one field in the periventricular white matter and the corona radiata of the first and second parasagittal gyrus (Fig. 1). In addition, one field in the lateral cortex and upper white matter tract of the first and second parasagittal gyrus was used to assess Olig2-, CNPase- and MBP-labelling. All images were quantified in ImageJ (National Institutes of Health, USA). The Olig2-, Ki67- and Caspase3-positive cells were counted, and area fraction was calculated for CNPase, MBP, GFAP and Iba1 immunolabelled sections, using default thresholding. Scores from two adjacent tissue sections and both hemispheres were averaged for each region. Results from the first and second parasagittal gyrus were combined for the corona radiata and superior white matter tract, as these regions were not significantly different (N.S.). Activated Caspase3-positive cells were identified as pyknotic cells with condensed nuclei.

### Tract direction calculation

The direction of CNPase- and MBP-labelled white matter in the parasagittal corona radiata was assessed with the OrientationJ plugin (Distribution) for ImageJ.^26^ This software determines the pixel orientation in the digital image using the structure tensor of a Gaussian-weighted (σ = 5 pixels) local area of interest. Local orientations (−89 to 89°) in each image are expressed in a frequency histogram, with pixel counts per angle of orientation calculated as a percentage area under the curve, subtracting the minimum percentage to reduce noise. The angle with the highest percentage of area under the curve is calibrated as 0°. The percentages of area under the curve were summated from angles −5 to 5°, based on the predominant tract direction observed in sham-ischaemia animals.

### Statistical analysis

Sham-ischaemia was compared to ischaemia-vehicle by ANOVA, with time or region treated as a repeated measure. With an estimated pooled SD of 80 for oligodendrocyte numbers from previous studies, *n* = 8 offered 80% power to detect an increase in oligodendrocyte number of 115 or more after each intervention compared to ischaemia alone. Histological analysis was performed by blinded observers (A.C., K.Q.Z. and S.K.D.). The experiments and statistical analysis were not blinded. The effect of two interventions was then assessed with mixed design ANOVA, including hypothermia and rEpo treatment as independent factors and time or anatomical region as repeated measures (SPSS v26, SPSS Inc., Chicago, IL, USA). When an overall effect of group or interaction between intervention, time or region was found, groups were compared by univariate ANOVA followed by the Sidak post-hoc test. Data are presented as mean (SD) for biological, physiologic and histological results. The Kruskal–Wallis test was conducted for non-parametric data. Significance was accepted at *P* < 0.05.

### Data availability

Data are available from the authors on reasonable request.

## Results

### Fetal biological data, plasma erythropoietin, and brain and core temperatures

There was no apparent difference in the proportion of singleton to twin pregnancies, fetal sex or weight between treatment groups at post-mortem (Table 1). Cerebral ischaemia was associated with reduced brain weight compared to sham-ischaemia (*P* = 0.003) that was improved with hypothermia (hypothermia versus normothermia groups, *P* = 0.007), but not rEpo treatment. rEpo infusion resulted in increased erythropoietin levels in arterial plasma (Table 1). Plasma erythropoietin levels were not different between ischaemia-Epo and ischaemia-Epo-hypothermia animals, and were below the detection limit in foetuses that did not receive infusion with rEpo. Hypothermia was associated with reduced extradural temperature to a mean of 32.0 ± 0.8°C within 3 h in the hypothermia groups, with no difference between the hypothermia groups, compared to 39.5 ± 0.3°C in the normothermia groups (*P* = 0.000 from 3 to 72 h, Table 1). Core temperature fell to a mean of 38.2 ± 0.3°C in the hypothermia groups, compared with 39.5 ± 0.3°C in the normothermia groups (*P* = 0.000 from 3 to 72 h, Table 1).

**Table 1:** Fetal biological parameters

Group	*N*	Foetus (g)	Brain (g)	Extradural temperature (C)	Core temperature (C)	Total rEpo AUC kIU/L*h	Singles/ Twins	Female/Male
Sham-Isch	9	4930 ± 824	48.9 ± 5.8	39.530.31	39.460.30	–	8:1	4:5
Isch-Veh	8	4575 ± 880	40.1 ± 4.3*	39.440.26	39.450.38	–	7:1	5:3
Isch-Epo	8	4411 ± 566	39.4 ± 3.4	39.570.25	39.430.27	6719 ± 1503	5:3	6:2
Isch-Hypo	8	4721 ± 412	44.9 ± 2.6$	33.391.45$	38.160.32$	–	8:0	4:4
Isch-Epo-Hypo	8	4662 ± 466	41.6 ± 2.9$	33.451.29$	38.250.23$	5777 ± 874	8:0	6:2

Fetal parameters for sham-ischaemia (sham-isch), ischaemia-vehicle (Isch-Veh), ischaemia-Epo (Isch-Epo), ischaemia-hypothermia (Isch-Hypo) and ischaemia-Epo-hypothermia (Isch-Epo-Hypo) animals. Fetal brain weight was reduced in ischaemia-vehicle animals (*P* < 0.05 versus sham-ischaemia), hypothermia had a significant effect on brain weight and temperatures and rEpo infusion increased plasma rEpo AUC; there was no significant effect of hypothermia on rEpo AUC. AUC was not calculated for animals that did not receive rEpo. Epo, erythropoietin; AUC, area under the curve. Data are presented as mean ± SD. *P < 0.05, ischaemia-vehicle versus sham-ischaemia; $*P* < 0.05 effect of hypothermia; #*P* < 0.05 effect of rEpo.

### Immunohistological outcomes

**Figure 2:**
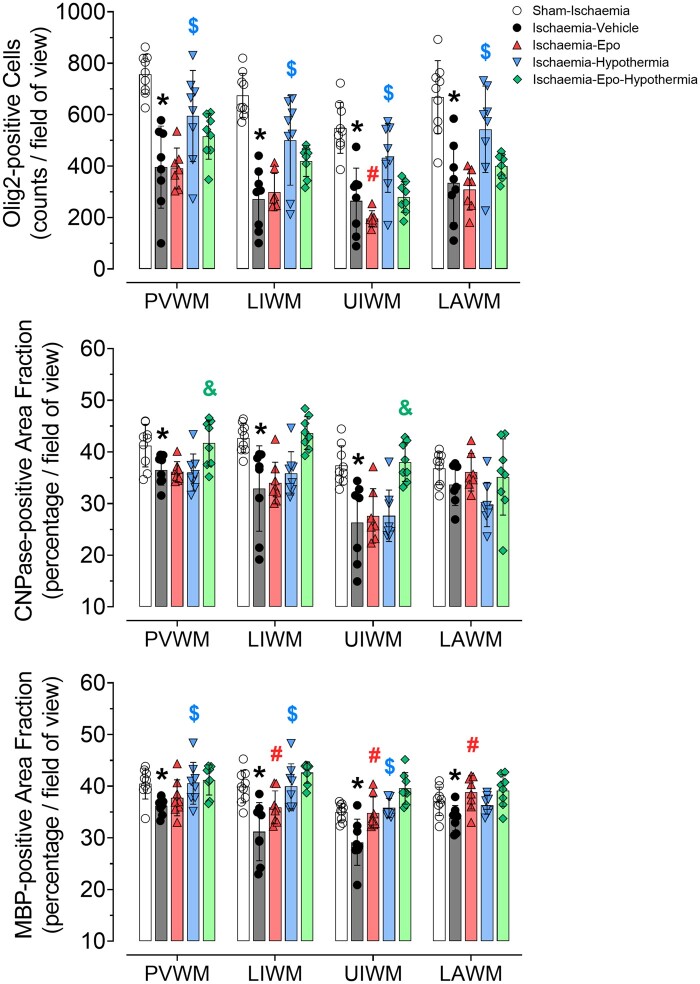
**Changes in total oligodendrocytes, CNPase and myelin basic protein.** Total oligodendrocytes (Olig2, *top panel*), CNPase (2′,3′-Cyclic-nucleotide 3′-phosphodiesterase, *middle panel*) and myelin basic protein (MBP, *bottom panel*) in the periventricular white matter (PVWM), lower intragyral white matter (LIWM) and upper intragyral white matter (UIWM) of the first and second parasagittal gyri, and the lateral white matter (LAWM) of sham-ischaemia (white circles, *n* = 9), ischaemia-vehicle (black circles, *n* = 8), ischaemia-Epo (red triangles, *n* = 8), ischaemia-hypothermia (blue triangles, *n* = 8), and the ischaemia-Epo-hypothermia (green diamonds, *n* = 8) groups at 7 days after cerebral ischaemia. Data are presented as mean ± SD. The sham control and ischaemia groups were compared using 2-Factor ANOVA with ischaemia as an independent variable and region as a repeated measure. The ischaemia groups were compared using 3-Factor ANOVA with hypothermia and rEpo as independent variables, and brain region as a repeated measure. *P < 0.05 versus sham-ischaemia; ^#^*P* < 0.05 effect of rEpo; ^$^*P* < 0.05 effect of hypothermia; ^&^*P* < 0.05 interaction between rEpo and hypothermia.

Ischaemia-vehicle was associated with significant loss of Olig2-positive cells and MBP-positive myelination in periventricular, parasagittal and lateral white matter (2-Factor ANOVA, *P* = 0.000, Fig. 2), compared with the sham-ischaemia group. Mild hypothermia improved overall Olig2-positive oligodendrocyte survival (*P* = 0.001), and restored MBP-positive area fraction in periventricular and parasagittal white matter tracts [2-Factor ANOVA; *F*(1) = 12.219, *P* = 0.002, and 3-Factor ANOVA; *F*(1) = 32.555, *P* = 0.000, Fig. 2], whereas rEpo treatment had no beneficial effect on total Olig2-positive oligodendrocytes, but restored MBP-positive myelin in parasagittal and later white matter [3-Factor ANOVA; *F*(1) = 12.690, *P* = 0.001, and 2-Factor ANOVA; *F*(1) = 16.827, *P* = 0.002]. There was no significant interaction between rEpo and hypothermia in any brain region for total Olig2-positive oligodendrocytes or MBP-positive myelination. Ischaemia-vehicle was associated with a reduction in CNPase positive myelin in the periventricular and parasagittal white matter tracts [1-Factor ANOVA; *F*(1) = 7.297, *P* = 0.016, and 2-Factor ANOVA; *F*(1) = 12.854, *P* = 0.003, Fig. 2] compared to sham-ischaemia animals, which was not improved with hypothermia or rEpo treatment. However, there was a significant interaction between hypothermia and rEpo, such that CNPase-positive myelination was improved compared to sham-ischaemia values in the periventricular and upper parasagittal white matter [2-Factor ANOVA; *F*(1) = 6.419, *P* = 0.017, and *F*(1) = 4.982, *P* = 0.034, Fig. 2].

**Figure 3:**
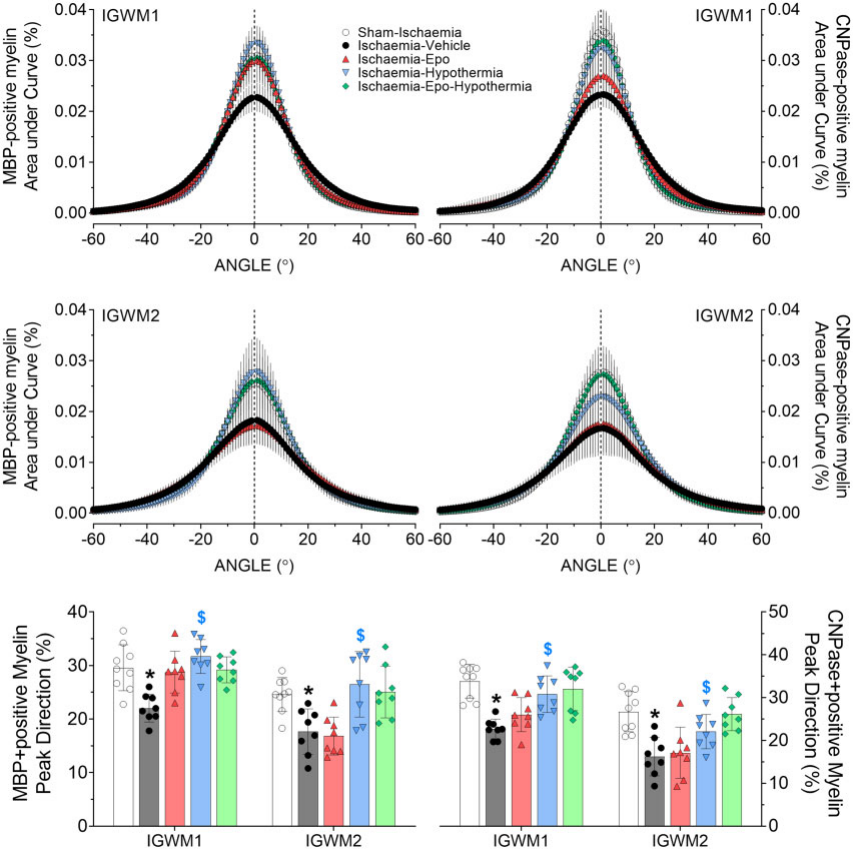
**Changes in myelin linearity of intra-gyral white matter.** Directionality of the myelin basic protein (MBP)- and 2′,3′-Cyclic-nucleotide 3′-phosphodiesterase (CNPase)-labelled myelin in the lower first and second intragyral white matter tracts (IGWM 1 and 2, *top and middle panels*) of the sham-ischaemia (white circles), ischaemia-vehicle (black circles, *n* = 9), ischaemia-Epo (red triangles, *n* = 8), ischaemia-hypothermia (blue triangles, *n* = 8), and ischaemia-Epo-hypothermia (green diamonds, *n* = 8) groups at 7 days after global cerebral ischaemia. Ischaemia reduced the linearity of myelin, with a lower percentage of MBP- and CNPase-myelin aligned to peak direction from −5 to +5° (*bottom panel*). Data are presented as mean ± SD. Analysis by 1-Factor ANOVA with ischaemia treated as an independent variable, and 3-Factor ANOVA with hypothermia and rEpo as independent variables, and regions treated as a repeated measure. *P < 0.05 versus sham-ischaemia; ^$^*P* < 0.05 effect of hypothermia.

**Figure 4:**
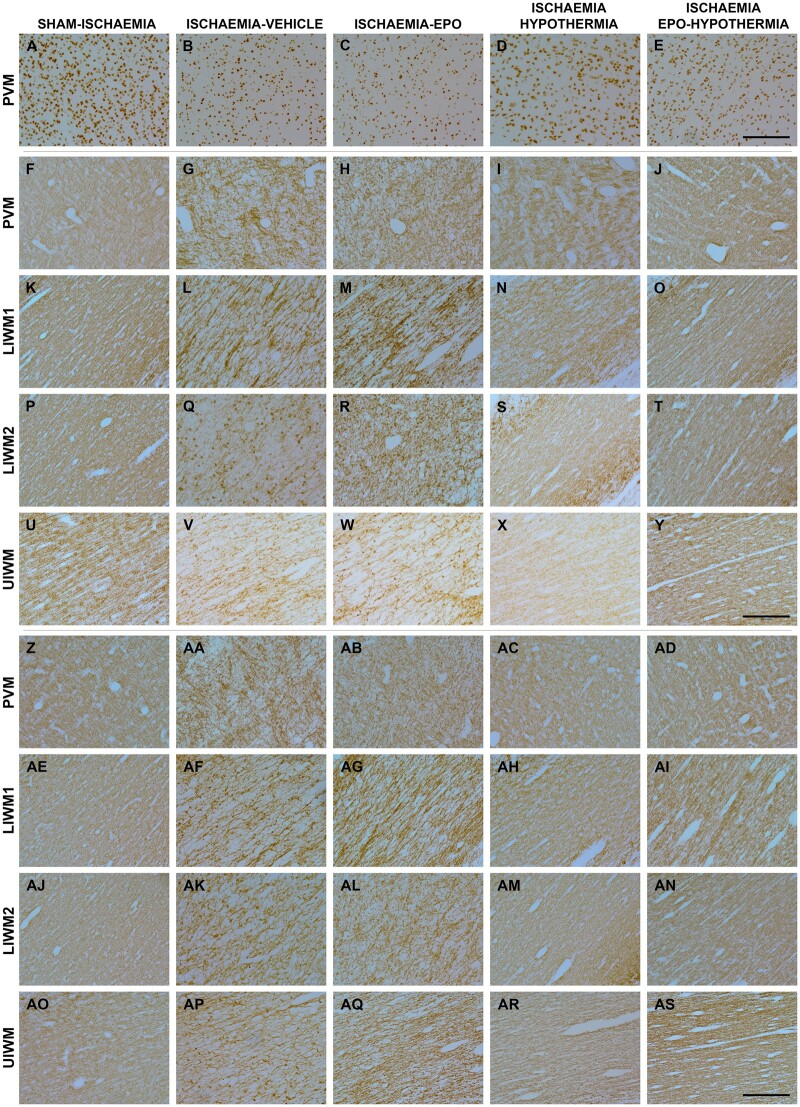
**Photomicrographs of total oligodendrocytes, CNPase and myelin basic protein.** Photomicrographs of total oligodendrocytes (Olig2-positive cells, panels **A–E**), and 2′,3′-Cyclic-nucleotide 3′-phosphodiesterase (CNPase)- (panels **F–Y**) and myelin basic protein (MBP)-labelled (panels **Z–AS**) myelin in the periventricular (PVM) and parasagittal intragyral white matter tracts of the sham-ischaemia, ischaemia-vehicle, ischaemia-Epo, ischaemia-hypothermia, and ischaemia-Epo-hypothermia groups at 7 days after cerebral ischaemia. Regions assessed included the corona radiata of the first and second parasagittal gyrus (lower intragyral white matter, LIWM 1 and 2) and the upper tract of the intragyral white matter (UIWM; first and second gyrus combined). Scale bar is 200 μm.

The direction of MBP- and CNPase-positive myelin was distributed within a narrow orientation range in the sham-ischaemia and ischaemia-Epo-hypothermia animals in the parasagittal white matter. Ischaemia-vehicle animals showed a broader distribution pattern, whereas ischaemia-Epo and ischaemia-hypothermia animals had a mixed outcome (Figs. 3 and 4). Ischaemia-vehicle reduced the overall proportion of MBP- and CNPase-positive myelin aligned to peak direction (−5 to 5°) in the parasagittal white matter tracts [2-Factor ANOVA; *F*(1) = 40.520, *P* = 0.000, and *F*(1) = 50.123, *P* = 0.000, Fig. 3]. Hypothermia restored the linearity of MBP myelin to sham-ischaemia values [3-Factor ANOVA; *F*(1) = 42.756, *P* = 0.000, Fig. 3], and partially improved CNPase myelin linearity [3-Factor ANOVA; *F*(1) = 31.827, *P* = 0.000]. Exploratory post-hoc testing showed a trend towards improved MBP myelin alignment in the ischaemia-Epo group in the first parasagittal white matter [2-Factor ANOVA; *F*(1) = 3.761, *P* = 0.063], with no significant interaction between rEpo and hypothermia.

**Figure 5:**
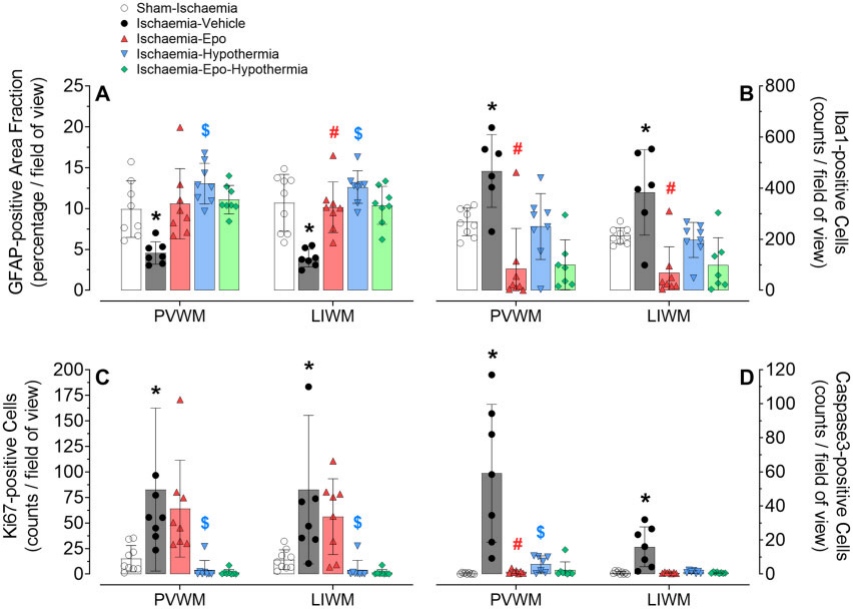
**Changes in astrocyte area fraction, microglia, proliferation, and apoptosis.** Astrocyte area fraction [glial fibrillary acidic protein (GFAP), panel **A**], and numbers of microglia [ionised calcium-binding adapter molecule-1 (Iba1), panel **B**], proliferating cells (Ki67, panel **C**), and apoptotic cells (caspase3, panel **D**) in periventricular (PVWM) and parasagittal lower intragyral white matter (LIWM; first and second gyri combined) of the sham-ischaemia (white circles, *n* = 9), ischaemia-vehicle (black circles, *n* = 8), ischaemia-Epo (red triangles, *n* = 8), ischaemia-hypothermia (blue triangles, *n* = 8), and ischaemia-Epo-hypothermia (green diamonds, *n* = 8) groups at 7 days after cerebral ischaemia. Data are presented as mean ± SD. Analysis by 2-Factor ANOVA with ischaemia as an independent variable and region as repeated measure, and 3-Factor ANOVA with hypothermia and rEpo treatment as independent variables, and region treated as a repeated measure. *P < 0.05 versus sham-ischaemia; ^#^*P* < 0.05 effect of rEpo; ^$^*P* < 0.05 effect of hypothermia.

**Figure 6:**
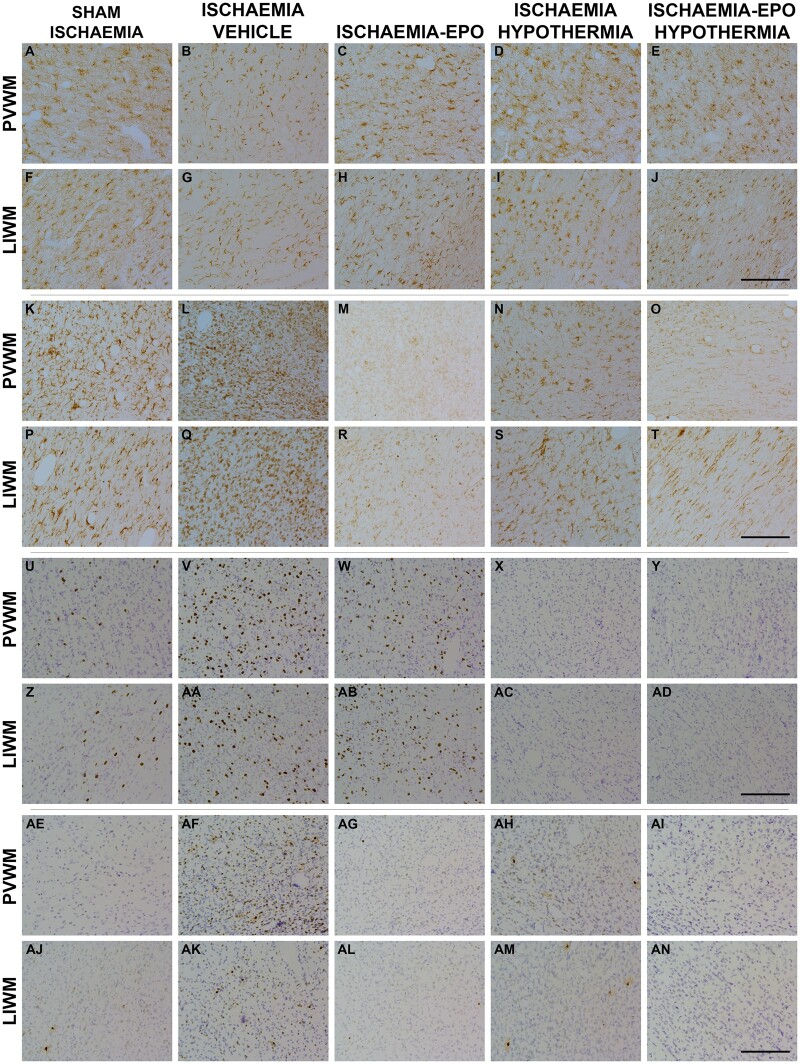
**Photomicrographs of astrocytes, microglia and proliferating and apoptotic cells.** Photomicrographs of astrocytes (panels **A–J**), microglia (panels **K–T**), and proliferating (panels **U–AD**) and apoptotic cells (panels **AE–AN**) in the PVWM and LIWM of the sham-ischaemia (column 1), ischaemia-vehicle (column 2), ischaemia-Epo (column 3), ischaemia-hypothermia (column 4) and ischaemia-Epo-hypothermia (column 5) groups at 7 days after cerebral ischaemia. Epo, erythropoietin. Scale bar is 200 μm.

Ischaemia-vehicle was associated with a significant reduction in GFAP-positive area fraction in the periventricular and lower parasagittal white matter [2-Factor ANOVA; *F*(1) = 24.961, *P* = 0.000, Figs. 5 and 6] compared with sham-ischaemia, and was restored by hypothermia or rEpo treatment [3-Factor ANOVA; *F*(1) = 29.192, *P* = 0.000, and *F*(1) = 5.875, *P* = 0.022]. In animals that received ischaemia-vehicle, there was a significant overall increase in Iba1-labelled microglia [2-Factor ANOVA; *F*(1) = 13.125, *P* = 0.003], Ki67-positive proliferating cells [2-Factor ANOVA; *F*(1) = 0.012, *P* = 0.012], and caspase3-positive cells [2-Factor ANOVA; *F*(1) = 7.435, *P* = 0.016] in periventricular and parasagittal white matter, compared to sham-ischaemia animals (Figs. 5 and 6). rEpo infusion resulted in a significant overall reduction in activated microglial cells [3-Factor ANOVA; *F*(1) = 28.484, *P* = 0.000] and apoptotic cleaved caspase3-positive cells [3-Factor ANOVA; *F*(1) = 5.973, *P* = 0.022], but had no effect on cell proliferation in these white matter regions. In the hypothermia-treated groups, there was overall suppression of Ki67-proliferating cells [3-Factor ANOVA; *F*(1) = 22.313, *P* = 0.000] and cleaved caspase3 cells [3-Factor ANOVA; *F*(1) = 5.010, *P* = 0.034], but not microglia (versus normothermia groups, Figs. 5 and 6). Post-hoc analyses suggested a trend towards a reduction in microglia [2-Factor ANOVA; *F*(1) = 4.110, *P* = 0.053] and fewer apoptotic caspase3 cells [2-Factor ANOVA; *F*(1) = 5.423, *P* = 0.028] in the periventricular, but not parasagittal, white matter tracts. There was no significant interaction between hypothermia and rEpo in these cell-types and regions.

## Discussion

This study demonstrates in term-equivalent fetal sheep that infusion with high-dose rEpo, from 3 to 72 h after severe cerebral ischaemia, was associated with MBP-myelin preservation in the parasagittal and lateral white matter, with attenuation of apoptosis and microgliosis after 7 days recovery. Combined treatment with high-dose rEpo and mild hypothermia improved CNPase-myelination in the periventricular and parasagittal white matter tracts, compared with hypothermia alone, but had no other additive effects. Thus, delayed, prolonged rEpo infusion and cerebral cooling provide independent white matter protection, with greater benefit from hypothermia. However, co-treatment had little additive effect, consistent with partially overlapping mechanisms of protection.

### rEpo treatment after cerebral ischaemia

Perinatal HI remains significantly associated with neonatal death and encephalopathy, and chronic neurodevelopmental impairment in infants born at term. Consistent with previous reports, cerebral ischaemia in this study was associated with overall loss of total numbers of oligodendrocytes, astroglial expression, and MBP- and CNPase myelin expression and tract linearity in the white matter, with greater cell proliferation, microglial activation and apoptosis.^26^^,^^27^ This histopathological phenotype is consistent with the white and grey matter damage typically observed in term infants with moderate-severe HIE, even after hypothermia treatment.^5^ Despite delaying treatment until 3 h post-ischaemia, a clinically plausible starting time,^28^ prolonged rEpo infusion improved overall MBP-labelled myelin and astroglial expression in white matter, in association with significant reductions in apoptosis and microgliosis. These anti-apoptotic and anti-inflammatory effects of rEpo treatment after HI are well-described and associated with neural and white matter protection and improved neurocognitive, EEG and behavioural function.^14^^,^^16^

Importantly, chronic myelin disruption and associated axonopathy are apparent contributors to poor neurodevelopment after HIE, as shown by consistent correlation between microstructural lesions in the PLIC and frontal and parietal white matter, and mental, language and locomotor impairments.^7^^,^^26^^,^^29^ Consistent with these reports, this study indicated modest reduction in MBP area fraction and structural tract integrity in parasagittal white matter at 7 days post-insult. Exploratory post-hoc tests suggested improvement to sham-ischaemic levels (*P* = 0.06) in the first, but not second, parasagittal gyrus of rEpo-treated foetuses. Of interest, Zhou et al.^26^ demonstrated a direct relationship between post-ischaemic restoration of MBP-myelin tract integrity, and reduction in axonal injury and neuronal loss in the parasagittal cortex. Encouragingly, improvement in MBP-myelination was associated also with partial parasagittal neuroprotection in this rEpo-treated cohort, as reported previously.^14^

HI induces sterile neuroinflammation that contributes to cortical demyelination and axonal damage via microglial release of free radicals and pro-inflammation cytokines such as interleukins-1ß, interleukin-6 and tumour necrosis factor.^30^^,^^31^ There is recent evidence that rEpo attenuates microglial hypertrophy and hyperplasia, and can polarize active cells from a pro-inflammatory M1 state, towards an anti-inflammatory M2 status after focal ischaemia and intracerebral haemorrhage in rodents.^32^^,^^33^ To the best of our knowledge, this is the first study to report that continuous rEpo (5000 IU/kg) infusion for 3 days after cerebral ischaemia is associated with potent suppression of microglial cells in white matter to less than sham-ischaemia levels, despite astrocytic restoration and no change in Ki67-positive cellular proliferation compared to ischaemia-vehicle. Consistent with previous studies,^32^^,^^33^ cerebral ischaemia resulted in a shift in the microglial phenotype from a (resting) ramified appearance to a predominantly amoeboid (activated) state. Interestingly, the microglia that remained in rEpo-treated animals mostly had an amoeboid phenotype. Microglial phenotype is highly labile, and knowledge of their relationship between form and function is still incomplete.^34^ Nevertheless, this finding suggests a disproportionate reduction of (beneficial) M2-type cells using this rEpo protocol, which might have attenuated protection of white matter.^14^ Overall, there is limited evidence on the effect of high-dose rEpo on microglia response after HI, and this remains an important area of research.

Limited evidence from human clinical trials also suggests partial neuroprotection with delayed rEpo treatment after HI. For example, in term infants with mild-moderate HIE, high-dose rEpo (2500 IU/kg) injections started at 4–6 h after birth attenuated breakthrough seizures, and neurologic and developmental deficits at 6 months of age, but with no effect on brain damage on modern imaging, including focal white matter lesions.^35^ Similarly, a small trial in 100 infants with moderate-severe HIE reported that 500 IU/kg rEpo injections, started within 6 h post-birth and administered at 48 h intervals for 5 doses total, improved brain abnormalities on magnetic resonance imaging and death or disability amongst infants with moderate, but not severe HIE.^36^ Finally, a recent large randomized, controlled trial of high dose rEpo given within 24 h of birth in extremely preterm infants showed no effect on neurodevelopmental outcomes at 2 years of age.^37^ In that trial, rEpo 1000 IU/kg was given i.v. every 48 h for a total of six doses, followed by 400 IU/kg three times per week by subcutaneous injection until 32 weeks postmenstrual age. The reasons for the lack of effect are not clear, but may include that treatment was started relatively late after birth, and that the intermittent injections were associated with prolonged periods with very low plasma rEpo levels, as recently demonstrated in the fetal sheep.^38^

As this trial highlights, the best rEpo regimen for cerebral protection after HI is still unclear. In neonatal p7 rats, therapeutic benefit was lost when single dosing with 1000 IU/kg rEpo was delayed until 1–3 h after HI, whereas a single high-dose rEpo (5000 IU/kg) injection at 6 h post-insult was still partially protective in juvenile rats with focal stroke.^39^^,^^40^ Consistent with this, one injection with 5000 IU/kg immediately after reperfusion was more protective than 2500 IU/kg, and three injections (at 24, 48 and 72 h post-insult) resulted in greater neuroprotection than a single dose.^41^

For comparison, in term fetal sheep, a single i.v. injection with 5000 IU/kg rEpo was associated with neuroprotective cerebrospinal fluid levels after 2.5 h.^42^ This dose was followed by a continuous rEpo infusion in this study, and led to a sustained increase in plasma rEpo concentration for three days.^14^ It is important to appreciate that this regime delivers a larger dose of rEpo in absolute terms than reported in clinical studies, because of the much greater clearance and volume of distribution in fetal sheep compared to infants with HIE.^21^

### Therapeutic hypothermia after cerebral ischaemia

In this study, prolonged cerebral hypothermia, from 3 to 72 h after global cerebral ischaemia, improved overall numbers of total Olig2-positive oligodendrocytes, and restored astroglia and density and linearity of MBP-myelin to sham-ischaemia levels in the periventricular and cortical white matter. This improvement was associated with attenuation of caspase-mediated apoptosis and the post-ischaemic increase in cell proliferation in white matter, indicating that hypothermia predominantly prevented cell death rather than enabling restorative oligodendroglial proliferation. Persistent inflammation has been reported after post-insult cooling.^43^ Critically, the current data show no difference in microglial cell numbers between ischaemia-hypothermia and sham-ischaemia (Fig. 5), although it is important to stress that both groups had marked within-group variation. Post-cooling microglia also had a predominantly ramified appearance. This would be consistent with previous evidence that hypothermia has a differential effect on the glial reaction to HI,^44^ with potent microglial modulation but minor effect on astroglia. This suggests that hypothermic white matter protection results, in part, from reducing inflammation post-ischaemia while not suppressing ‘beneficial’ glial processes.

Interestingly, hypothermia improved CNPase linearity in the parasagittal white matter, but had no significant effect on CNPase density. The reason for this differential outcome is unclear. In post-ischaemic fetal sheep, Roelfsema et al. demonstrated that hypothermia for 72 h protected MBP labelling and numbers of proteolipid protein-labelled oligodendrocytes in parasagittal white matter when initiated at 1.5 h after cerebral ischaemia. However, this protection was lost for proteolipid protein-labelled, but not MBP-labelled, oligodendrocytes when hypothermia was delayed until 5.5 h post-ischaemia.^10^ By contrast, there was still significant grey matter protection in the parasagittal and lateral cortex when cooling was begun at 5.5 h after reperfusion.^9^ Thus, it is reasonable to consider that the ‘therapeutic window’ for treatment is likely to be cell-type specific and may be shorter for oligodendroglia.

### rEpo plus hypothermia after cerebral ischaemia

The present observation that delayed cerebral hypothermia protected white matter more than high-dose rEpo started at the same time and continued for the same duration confirms a previous small cohort (*n* = 20) trial in term neonates with HIE that systemic cooling was more neuroprotective than a single subcutaneous injection with 1500 IU/kg of rEpo.^45^ Critically, in this study, co-treatment with hypothermia plus rEpo improved CNPase to sham-ischaemia levels in the periventricular and parasagittal white matter, whereas independent rEpo or hypothermia treatment did not improve this histological outcome. It is possible that co-treatment was associated with synergic protective effects that prevented the myelinating cells from committing to cellular death, despite there being no difference in astrocytic, microglial or caspase-3 responses compared to hypothermia alone.

Hypothermic protection is associated with suppression of metabolic and pathologic processes,^46^ which potentially could extend the effective treatment window of rEpo. There is some, limited evidence to support this. For example, in adult rats with forebrain stroke, intracortical injection with maslinic acid before HI prolonged the time for neuroprotection and infarct volume reduction with dizocilpine from 1 to 3 h post-insult.^47^ Similarly, in adult rats subjected to HI, when subjects were cooled immediately after HI, neuroprotection with exogenous IGF-1 could be maintained despite extending the delay in administration from 2 to 6 h post-insult.^48^ Nevertheless, in this study, improvement in CNPase-myelin area fraction with co-treatment was not associated with greater myelin linearity, and thus overall combined treatment had limited benefit compared to therapeutic hypothermia alone.

Our findings are broadly consistent with the limited number of preclinical studies that tested co-treatment of hypothermia with rEpo. For example, in neonatal rats with HI, treatment with rEpo plus systemic cooling did not reduce white matter lesions or motor impairment, compared to hypothermia alone.^17^ In non-human primates asphyxiated at birth, repeated rEpo doses (at 24, 48 and 72 h) plus whole-body cooling improved fractional anisotropy values in the corpus callosum and internal capsule on diffusion tensor imaging;^18^^,^^19^ but comparison to hypothermia was impossible due to limited animal numbers. One phase II (*n* = 50) double-blind randomized trial in infants with moderate-severe HIE of hypothermia plus repeated rEpo dosing (1000 IU/kg given 1, 2, 3, 5 and 7 days after birth) has been published.^21^ This trial reported lower global brain damage scores in rEpo-treatment neonates (median, 2 versus 11, *P* = 0.01), with reduced injury in subcortical regions (*P* = 0.02, including the posterior limb of the internal capsule), but no change in the white matter or cortex (median 12 versus 15; *P* = 0.80, and median 4 versus 9; *P* = 0.26, respectively) on MRI at 5.1 days. Neurological follow-up assessments at 12.7 months of age showed an apparent trend to improved motor scores (Alberta Infant Motor Scale, *P* = 0.09) but no effect on the Warner Initial Developmental Evaluation scores (*P* = 0.43). Results from larger phase-III clinical trials are awaited.

The most likely reason for lack of benefit with combined therapy in this study is that rEpo and hypothermia had overlapping anti-apoptotic and anti-inflammatory effects as shown by reduced activated caspase-3 and Iba-1 positive microglial expression, similarly to previous studies.^12–14^ Potentially, white matter protection though these mechanisms may have been maximal after therapeutic hypothermia and so could not be augmented by add-on rEpo.^46^ Alternatively, it is striking that rEpo did not affect the marked Ki67-positive proliferation at day 7 after ischaemia, whereas hypothermia suppressed proliferation and proliferation remained suppressed after combined treatment. Hypothermia consistently reduces activation of many intracellular pathways,^46^ whereas rEpo provides anti-apoptotic, anti-inflammatory and pro-proliferative effects by activating the Epo receptor on brain cells.^13^ Thus, it is possible that hypothermia during exogenous treatment with rEpo actively reduced the neuroprotective effects of rEpo.

In this context, it is interesting to reflect on the increasing evidence that brain injury may continue to evolve for weeks, months and even longer after perinatal HI through programmed cell death and inflammatory mechanisms.^49^ This suggests the intriguing possibility that treatment with rEpo started *after* rewarming from therapeutic hypothermia, at a time when there would be no hypothermic suppression of its effects, might promote neural restoration.^12^^,^^13^

In conclusion, this study using a well-established large animal model of global cerebral ischaemia demonstrates that delayed infusion of high-dose rEpo from 3 to 72 h post-ischaemia was associated with partial white matter protection, but that co-treatment did not materially improve hypothermic white matter protection. We propose that the strategy of infusing rEpo *after* rewarming from therapeutic hypothermia should be investigated in future studies.

## Funding

This research received financial support from the Health Research Council of New Zealand (17/601), the Neurological Foundation of New Zealand (1715-PG) and the Auckland Medical Research Foundation (1121001).

## Competing interests

The authors report no competing interests.

## Abbreviations

CNPase =: 2′,3′-cyclic-nucleotide 3′-phosphodiesterase
d =: days
GFAP =: glial fibrillary acidic protein
HI =: hypoxia-ischaemia
HIE =: hypoxic-ischaemic encephalopathy
Iba1 =: ionized calcium-binding adapter molecule-1
i.v.: intravenous
MAP =: mean arterial blood pressure
MBP =: myelin basic protein
NGS =: normal goat serum
Olig2 =: oligodendrocyte transcription factor
rEpo =: recombinant erythropoietin
